# Previous cocaine self-administration disrupts reward expectancy encoding in ventral striatum

**DOI:** 10.1038/s41386-018-0058-0

**Published:** 2018-04-10

**Authors:** Amanda C. Burton, Gregory B. Bissonette, Daniela Vazquez, Elyse M. Blume, Maria Donnelly, Kendall C. Heatley, Abhishek Hinduja, Matthew R. Roesch

**Affiliations:** 1 Department of Psychology, 1147 Biology-Psychology Building University of Maryland, College Park, MD 20742 USA; 2 Program in Neuroscience and Cognitive Science, 1147 Biology-Psychology Building University of Maryland, College Park, MD 20742 USA

## Abstract

The nucleus accumbens core (NAc) is important for integrating and providing information to downstream areas about the timing and value of anticipated reward. Although NAc is one of the first brain regions to be affected by drugs of abuse, we still do not know how neural correlates related to reward expectancy are affected by previous cocaine self-administration. To address this issue, we recorded from single neurons in the NAc of rats that had previously self-administered cocaine or sucrose (control). Neural recordings were then taken while rats performed an odor-guided decision-making task in which we independently manipulated value of expected reward by changing the delay to or size of reward across a series of trial blocks. We found that previous cocaine self-administration made rats more impulsive, biasing choice behavior toward more immediate reward. Further, compared to controls, cocaine-exposed rats showed significantly fewer neurons in the NAc that were responsive during odor cues and reward delivery, and in the reward-responsive neurons that remained, diminished directional and value encoding was observed. Lastly, we found that after cocaine exposure, reward-related firing during longer delays was reduced compared to controls. These results demonstrate that prior cocaine self-administration alters reward-expectancy encoding in NAc, which could contribute to poor decision making observed after chronic cocaine use.

## Introduction

The nucleus accumbens core (NAc) region of the ventral striatum (VS) is thought to be critical for the integration of limbic and motor information during reward-based learning and decision making [1–3]. One mechanism by which NAc serves this function is to signal the value of expected outcomes during reward anticipation. It has been shown that neurons in NAc fire after an instrumental response or presentation of a Pavlovian cue as animals anticipate the imminent delivery of reward [4–16]. This signal is thought to play a critical role in timing rewarded outcomes during behavior. Consistent with these neural correlates, several studies have shown that perturbation of NAc impacts functions related to reward timing and performance on temporal discounting tasks [17–28] and alters neural signaling in downstream regions such as the ventral tegmental area (VTA) and dorsal lateral striatum (DLS) during similar tasks [28, 29].

Taken together, these studies suggest that the NAc plays a critical role in guiding normal decision making by integrating and providing information to downstream areas about the timing and value of obtained and expected reward. Importantly, this function is thought to be altered after chronic cocaine exposure; rats exposed to cocaine are more sensitive to delays to reward, impulsively choosing more immediate reward more often than delayed reward relative to controls [30–34]. Although it is clear that NAc signals reward expectancies that are critical for timing of outcomes and normal delay discounting behavior, and that cocaine exposure impacts NAc and heightens impulsivity, we still do not know how expectancy correlates are disrupted after chronic drug use.

To address this issue, we recorded from single neurons in the rat NAc after previous sucrose (control) or cocaine self-administration. Neural recordings were taken while rats performed an odor-guided decision-making task in which we independently manipulated value of expected reward by changing the delay to or size of reward across a series of trial blocks. We found that previous cocaine exposure increased behavioral biases toward immediate reward on free-choice trials and decreased performance on forced-choice trials compared to controls. In addition, cocaine-exposed rats showed decreased counts of neurons responsive to odor cues and rewards, and those that remained responsive exhibited diminished value and directional encoding within the NAc at the time of reward compared to controls.

## Materials and methods

### Subjects

Ten male Long-Evans rats were obtained at 175–200 g from Charles River Laboratories. Rats were tested at the University of Maryland, College Park in accordance with UMD and NIH guidelines.

Odor-guided delay/size choice task. Before surgery, all rats were trained on the odor-guided delay/size choice task for ~6 weeks (for more detail see ref. [35]). On each trial, nose poke into the odor port after house light illumination resulted in delivery of a directional odor cue. One of three different odors (2-Octanol, Pentyl Acetate, or Carvone) was pseudorandomly delivered to the port on each trial. One odor instructed the rat to go to the left fluid well to receive reward (forced-choice), a second odor instructed the rat to go to the right fluid well to receive reward (forced-choice), and a third odor indicated that the rat could obtain reward at either well (free-choice). On forced-choice trials, if the rat went to the incorrect well, reward was not delivered. Odors were presented in a pseudorandom sequence, such that the free-choice odor was presented on 7/20 trials and the left/right odors were presented in equal proportions. In addition, the same odor could be presented on no more than three consecutive trials. Odors were counterbalanced across rats.

**Fig. 1:**
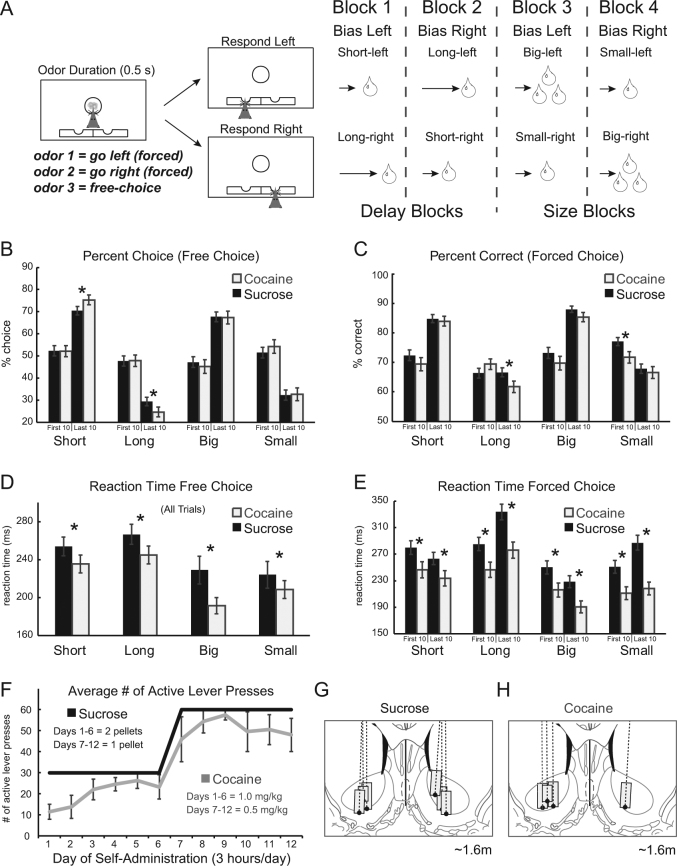
**a** Task schematic, showing sequence of events in one trial (left panels) and the sequence of blocks in a session (right). Rats were required to nose-poke in the odor port for 0.5 s before the odor turned on for 0.5 s instructing them to respond to the adjacent fluid wells below where they would receive liquid sucrose reward after 500–7000 ms. For each recording session, one fluid well was arbitrarily designated as short (a short 500 ms delay before reward) and the other designated as long (a relatively long 1–7 s delay before reward) (Block 1). After the first block of trials (∼60 trials), contingencies unexpectedly reversed (Block 2). With the transition to Block 3, the delays to reward were held constant across wells (500 ms), but the size of the reward was manipulated. The well designated as long during the previous block now offered an additional fluid bolus (i.e., large reward), whereas the opposite well offered 1 bolus (i.e., small reward). The reward stipulations again reversed in Block 4. **b** Percent choice on free-choice trials in each value manipulation over the first ten and last ten trials of each block averaged across animals and sessions (controls, black bars; cocaine, gray bars). **c** Percent correct on forced-choice trials in the same manner as **b**. **d** Reaction time (odor port exit minus odor offset) on all free-choice trials for each value manipulation. **e** Reaction time (odor port exit minus odor offset) on forced-choice trials in the same manner as **b** and **c**. For these analyses, behavior was looked at by session. **f** Average number of lever presses during sucrose (black) or cocaine (gray) self-administration averaged across rats for each day (days 1–12). **g, h** Location of recording sites (Paxinos and Watson). Gray boxes mark the extent of the recording locations. Sucrose control group *n* = 6; cocaine group *n* = 4. Error bars indicate SEM. Asterisks (*) indicate significance (*p* < 0.05) in multi-factor ANOVA and/or post-hoc *t*-tests

At the start of each session, one well was randomly designated as short delay (500 ms) and the other long (1–7 s) (Fig. 1a: Block 1). In the second block of trials, these contingencies were switched (Fig. 1a: Block 2). The length of the delay under long conditions abided by the following algorithm: the side designated as long started with a delay of 1 s and increased by 1 s every time that side was chosen by the rat during a free-choice odor trial (up to a maximum of 7 s). The delay for forced-choice trials was yoked to the delay on free-choice trials. In later blocks, we held the delay preceding reward delivery constant (500 ms) while manipulating the size of the expected reward (Fig. 1a: Blocks 3 and 4). The reward was a 0.05 ml bolus of 10% sucrose solution. For big reward, an additional bolus was delivered 500 ms after the first bolus. At least 60 trials per block were collected for each neuron and session. Thus, rats chose either between short and long delay (first two blocks) or large and small reward (final two blocks). Size and delay variables were independently manipulated so that reward size and delay never simultaneously varied within the same block of trials. Each behavioral session contained four basic trial types (short, long, big, and small), two possible response directions (left and right), and two stimulus types (free- and forced-choice odors).

### Surgery, self-administration, and recording

All rats were implanted with catheters for self-administration and drivable electrodes (1.6 mm anterior to bregma, + or − 1.5 mm laterally, and 6 mm ventral to the brain surface) for single-unit recordings (sucrose control group, *n* = 6 and cocaine group, *n* = 4) as describe previously [35]. After rats recovered from surgery, a twelve-day self-administration protocol was implemented using Med Associates Inc. operant behavioral boxes [35]. During days 1–6, rats self-administered 1 mg/kg dosage of cocaine or two sucrose pellets (control) via lever press on a fixed-ratio one schedule; the session ended after either a maximum of 30 presses occurred or 3 h elapsed. During days 7–12, the dosage of cocaine was 0.5 mg/kg and only one sucrose pellet was delivered (FR1); the session ended after a maximum of 60 presses occurred or 3 h elapsed. The rationale for shifting from high to low doses was to show that rats sought the same level of cocaine when the dose was cut in half, allowing us to gage escalation of drug-seeking behaviors. Recordings began 1 month after self-administration so that we could examine long-term cocaine-induced changes in neural activity and behavior when no drugs were in the system (for further details see refs. [35–37]).

### Neural analysis

Extracted single units were sorted in Offline Sorter using template matching (Plexon) and exported to NeuroExplorer and Matlab. Unless otherwise stated, only correct rewarded trials were analyzed and both free- and forced-choice trials used. Three main analysis epochs were examined. One was taken 250 ms before reward delivery to 1 s after reward delivery (reward epoch) to capture activity related to expectancy and reward delivery. This 250 ms period was chosen because it does not impinge on early activity occurring as a result of odor response, even at the shortest delay of 500 ms before reward delivery. Another analysis epoch was taken 100 ms after odor onset to fluid-well entry (odor epoch). This period of time did not overlap with the reward epoch and captured activity before fluid-well entry. Both epochs were compared to baseline (1 s before odor onset; Wilcoxon; *p* < 0.05) to determine task responsiveness; baseline firing rate for control (7.49 spikes/s) and cocaine rats (7.72 spikes/s) did not significantly differ from each other (*t* test; *t*(1123) = 0.49; *p* = 0.62). Lastly, for analysis of long delays we also used an epoch starting 500 ms after fluid-well entry to capture initial bursts of activity that were elicited upon well entry. This epoch did not overlap with the reward epoch even at the shortest long-delay trial (1 s). Wilcoxon (*p* < 0.05) analyses were used to determine differences in firing at the single-neuron level. Chi-squares (*p* < 0.05) were performed on counts of neurons in each group to determine if there were any significant differences between groups. For each neuron, difference scores were computed between high and low value rewards (short−long/short + long and big−small/big + small) and correct and error trials (correct−error/correct + error). Wilcoxon (*p* < 0.05) tests were used to measure significant shifts from zero and to determine if these shifts significantly differed between groups.

## Results

### Self-administration

All rats were trained on the reward-guided decision-making task (Fig. 1a) prior to implantation of electrodes in VS (Fig. 1g, h) and catheters for cocaine self-administration (see Methods for more detail). Rats self-administered sucrose pellets or cocaine paired with a cue light over the course of 12 days. During days 1–6 (1 mg/kg cocaine or two sucrose pellets), the average number of active lever presses across rats out of a maximum of 30 was 20.2 (±9.8; standard deviation; s.d.) and 30 (±0 s.d.) for cocaine and sucrose, respectively (Fig. 1f). During days 7–12 (0.5 mg/kg cocaine or one sucrose pellet), the average number active lever presses across rats out of a maximum of 60 was 51 (±14.1 s.d.) and 60 (±0 s.d.) for cocaine and sucrose, respectively (Fig. 1f).

### Cocaine made rats more sensitive to delay manipulations

One month after self-administration we recorded from single units in the NAc from six sucrose control rats and four cocaine-exposed rats during performance of the odor-guided decision-making task. Behavior was looked at by session, with a total of 154 sessions from controls (20, 22, 21, 29, 25, and 37) and 95 sessions from cocaine rats (19, 25, 28, and 23). Multi-factor ANOVAs were performed to assess performance in the task for several behavioral measures including percent correct, percent choice, and reaction time (odor offset to odor port exit) on both forced- and free-choice trials. For this behavioral analysis, we used completed sessions as the sample size in order to better equate behavior to neural firing. Factors in the ANOVA included group (sucrose vs. cocaine), value (high vs. low), value manipulation (size vs. delay), and phase of learning (early: first ten trials vs. late: last ten trials).

Performance on free-choice trials was similar to previous results using the same task in that we see a bias toward higher value rewards during delay blocks [32, 35]. In the ANOVA with percent choice as the dependent variable and the factors described above, there was a significant main effect of value (*F*(1,1976) = 1025.4, *p* < 0.01), as both control and cocaine rats chose short-delay and large-reward more often than long-delay and small-reward, respectively (Fig. 1b). There was also a significant interaction between group, value and value manipulation (*F*(1,1976) = 1592.6, *p* < 0.01) and between group, value and phase (*F*(1,1976) = 1398.4, *p* < 0.01) with cocaine rats choosing the high-value reward significantly more often than controls in the last ten free-choice trials during delay manipulations (Fig. 1b; *t*(247) = 2.86, *p* < 0.01). As shown previously, cocaine-exposed rats were strongly drawn to the more immediate reward at the end of delay blocks [30–35]. Additionally, cocaine rats were significantly faster on all free-choice trial types compared to controls (Fig. 1d; ANOVA; main effect of group (*F*(1988) = 6.03, *p* < 0.05). Although cocaine made rats more sensitive to delay manipulations, cocaine and control rats chose large over small reward at similar rates (Fig. 1b; *t*(247) = 0.19, *p* = 0.844).

In separate ANOVAs with percent correct and reaction time on forced-choice trials as the dependent variables and the same factors as stated above, we found a main effect of value (percent correct: *F*(1,1976) = 201.66, *p* < 0.01; forced-choice reaction time: *F*(1,1976) = 29.01, *p* < 0.01) and an interaction of value and phase (percent correct: *F*(1,1976) = 199.08, *p* < 0.01; forced-choice reaction time: *F*(1,1976) = 28.41, *p* < 0.01). This indicates that overall, both control and cocaine rats were significantly better and faster on high value forced-choice trial outcomes particularly in the late phase of each block (Fig. 1c, e). However, there was also a main effect of group in the ANOVAs on forced-choice behavioral measures (percent correct: *F*(1,1976) = 10.65, *p* < 0.01; forced-choice reaction time: *F*(1,1976) = 83.91, *p* < 0.01), with cocaine rats being significantly faster and worse on forced-choice trials compared to controls (Fig. 1c, e).

We conclude that previous cocaine self-administration had a long-term impact on behavior during performance of the odor-guided decision-making task. Overall, cocaine rats exhibited stronger response biases toward more immediate reward on free-choice trials and were significantly faster and worse on forced-choice trials. These results are consistent with previous work demonstrating that cocaine self-administration makes rats more impulsive during delay trials [30–35].

### Previous cocaine self-administration alters reward-related activity in NAc compared to controls

**Fig. 2:**
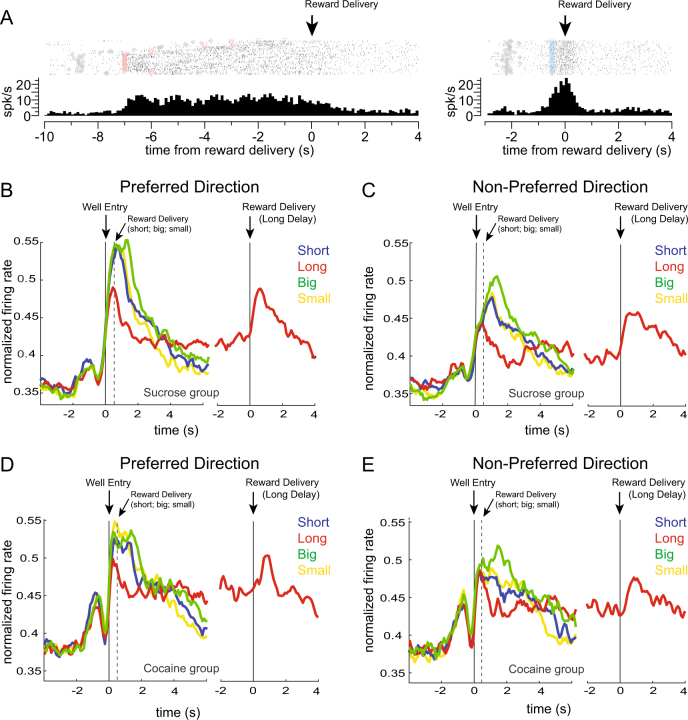
Previous cocaine-exposure diminishes value and directional encoding in NAc. **a** Single neuron example during long-delay (left panel) and short-delay trials (right panel). Activity is aligned to reward delivery (marked by arrows) and binned at 100 ms. One tick mark equals one action potential. **b, c** Population activity for responsive neurons in control rats (*n* = 195; 6 rats) during the last ten trials for each outcome. **b** Normalized firing rates for neurons in the preferred direction (direction that elicited the most activity). **c** Normalized firing rates for neurons in the non-preferred direction. Figure includes both forced and free-choice trials. Blue lines are short-delay trials, red lines are long-delay trials, green lines are large-reward trials, and yellow lines are small-reward trials. Activity is aligned to fluid-well entry for short, big and small trials (reward occurred 500 ms later; dashed gray line). For long-delay trials activity is split, aligned to “Well Entry” and “Reward Delivery”. **d, e** Same as **c**–**b** but for neurons (*n* = 103; 4 rats) collected from rats that had self-administered cocaine

In control and cocaine-exposed rats, 195 (30%) and 103 (21%) neurons increased firing during the reward epoch (250 ms before reward delivery to 1 s after reward delivery) compared to baseline (1 s before odor onset; Wilcoxon, *p* < 0.05), respectively. The frequency of responsive neurons was significantly different between groups, with more neurons in NAc in controls significantly increasing firing during the reward epoch compared to cocaine rats (*χ*^2^ = 6.47, *p* < 0.05). In Fig. 2a, we show a single neuron example of anticipatory activity during performance of a long delay trial, where firing to delayed rewards remained elevated until reward delivery (left panel). On short delay trials (right panel), the same neuron was also active across the delay period (500 ms), but firing was stronger after shorter delays, peaking at the time of reward delivery.

To further examine the effects of reward on neural activity in the NAc, we plotted the normalized average population activity for all neurons that increased activity during the last ten trials of each block; firing was aligned to fluid-well entry for all trial types and to reward-delivery on long-delay trials (Fig. 2b–e). Since NAc neurons are known to be directionally tuned (i.e., fire more strongly for one movement direction relative to the other [13]) without preference for one direction over the other across the entire population of neurons, we determined which direction elicited the most activity on rewarded trials and designated this as the preferred direction (Fig. 2b, d, left panels) and the other direction as the non-preferred direction (Fig. 2c, e, right panels). In these plots, the colored lines reflect the neural response to the value of reward on that particular set of trials, and the delay is split on long delay trials so that activity can be aligned to both fluid-well entry and reward delivery. For the other trial-types—short, small, and large—the delay was a fixed 500 ms, thus well entry and reward delivery alignments were equivalent.

**Fig. 3:**
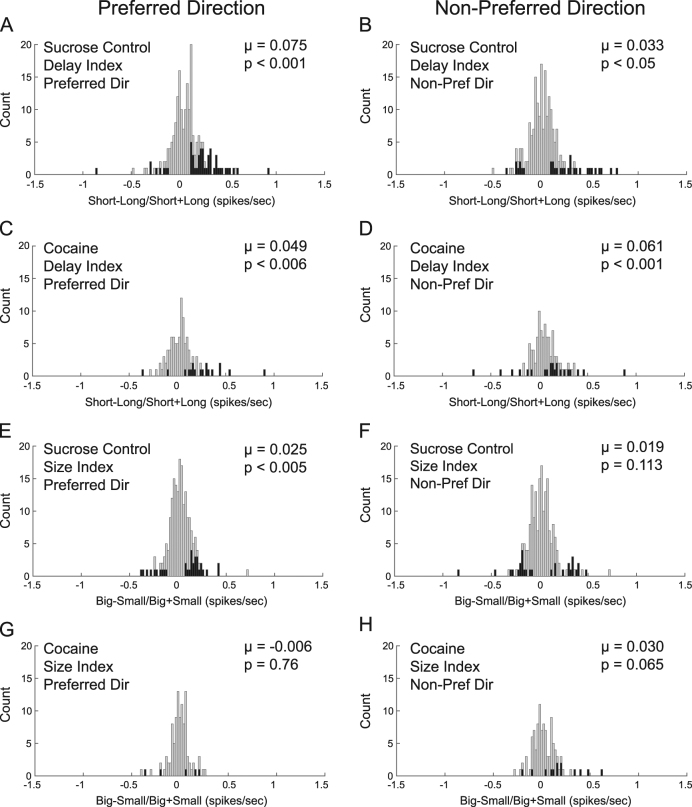
Distribution of value indices for delay (**a–d**) and size manipulations (**e–h**) for control (**a**, **b**, **e**, **f**; *n* = 195; 6 rats) and cocaine rats (**c**, **d**, **g**, **h**; *n* = 103; 4 rats) in the preferred (left panels) and non-preferred direction (right panels). Delay index = short−long/short + long during the reward-epoch; size index = big−small/big + small during the reward epoch. Distributions include both forced and free-choice trials. Neural activity was taken 250 ms before reward delivery to 1 s after reward delivery. Black bars represent neurons that showed significant modulation of expected outcome (Wilcoxon, *p* < 0.05)

As expected, activity in NAc in the preferred direction was modulated by value; there was an increase in firing for high-value outcomes compared to low-value outcomes (Fig. 2b; blue (short) and green (big) lines are higher than red (long) and yellow (small) lines, respectively). More specifically, firing was higher during large (green) compared to small (yellow) reward trials after the additional bolus was delivered. Also notable is that firing during reward delivered on short-delay (blue) and small-reward (yellow) trials were similar, consistent with both trial-types being physically the same (i.e., 1 bolus delivered after 500 ms delivery). Lastly, we see that firing was higher on short (blue) compared to long delay trials (red) both after well entry and after the long delay. In cocaine rats, value encoding was diminished in the preferred direction (Fig. 2d). To quantify this effect, we computed value indices on firing rates during the reward epoch by taking difference scores for each increasing-type neuron during each value manipulation (short−long/short + long and big−small/big + small). We found that control rats showed a significant positive shift from zero for both delay (Fig. 3a, *p* < 0.01, *µ* = 0.075) and size indices (Fig. 3e, *p* < 0.01, *µ* = 0.025), indicating that the majority of NAc neurons in control rats exhibited significantly increased activity for higher value outcomes (short and large reward, respectively) in the preferred direction. Like controls, in cocaine rats there was also a significant positive shift in the distribution of delay indices in the preferred direction (Fig. 3c, *p* < 0.01, *µ* = 0.049); however, unlike controls, there were no significant shifts from zero in the distribution of size indices (Fig. 3g, *p* = 0.76, *µ* = −0.0065), indicating that NAc neurons did not tend to fire more strongly for large compared to small reward after cocaine self-administration.

When directly comparing the delay and size index distributions between control and cocaine rats, we found that there were significant differences between groups (Wilcoxon rank sum, *p*’s < 0.05). The shifts above zero as discussed above in both the delay and size indices in the preferred direction for control rats was significantly stronger in controls compared to cocaine rats (Fig. 3, **a** versus **c** and **e** versus **g**; Wilcoxon rank sum, *p*’s < 0.05), indicating stronger value encoding in the NAc of control rats during both delay and size manipulations. Further, in size blocks, the variance significantly differed between distributions (preferred direction: *F*(102,194) = 0.66; *p* < 0.05; non-preferred direction; *F*(102,194) = 0.70; *p* < 0.05). On delay blocks, there were no significant differences between variances (*F*(102,194) = 0.77; *p* = 0.14; non-preferred direction; *F*(102,194) = 0.94; *p* = 0.72).

Comparing counts of single neurons that were significantly selective for size and delay (i.e., Fig. 3; black bars) further supports the finding that value selectivity was reduced after cocaine self-administration. To illustrate this, we performed a Wilcoxon test (*p* < 0.05) on firing rates for each individual neuron in the preferred and non-preferred direction and for each value condition in the task (short, long, big, small; black bars in Fig. 3a–h). In the preferred direction, control rats showed significant differences between counts of neurons for short compared to long delay (short *n* = 43, long *n* = 8, Fig. 3a, *χ*^2^ = 23.88, *p* < 0.01) and between counts of neurons for big compared to small reward (big *n* = 23, small *n* = 10, Fig. 3e, *χ*^2^ = 5.04, p < 0.05). Cocaine rats showed a similar pattern between counts of neurons in the preferred direction for short compared to long (short *n* = 16, long *n* = 2, Fig. 3c, *χ*^2^ = 8.76, *p* < 0.01). However, there were minimal counts of neurons in the preferred direction for big and small conditions, and the difference between counts was not significant in cocaine rats (big *n* = 3, small *n* = 2, Fig. 3g, *χ*^2^ = 0.162, *p* = 0.69).

In the NAc of control rats, neural activity reflects directional encoding of reward in a value-dependent manner. At the time of reward delivery, we see increased activity and increased counts of neurons responding to short compared to long delays and big compared to small rewards in the preferred direction. However, in cocaine rats this value encoding was diminished. Next, we consider firing in the non-preferred direction. In control rats, weaker value encoding was observed in the non-preferred direction for both delay (although still significant; Figs. 2c and 3b, *p* < 0.05, *µ* = 0.033) and size blocks (Figs. 2c and 3f, *p* = 0.113, *µ* = 0.019). In rats that had been exposed to cocaine, the opposite was true: cocaine rats showed significantly stronger value encoding in the non-preferred direction on delay blocks compared to the preferred direction (Fig. 3d, *p* < 0.05, *µ* = 0.061) and this effect was significantly stronger compared to controls (Wilcoxon rank sum, *p* < 0.05). In addition, cocaine rats showed stronger value encoding that approached significance in the non-preferred direction on size blocks compared to the preferred direction (Fig. 3h, *p* = 0.06, *µ* = 0.030). Thus, value encoding was stronger in the preferred direction compared to the non-preferred direction for both delay and size manipulations in controls, and this was not true for the cocaine group. In cocaine animals, value encoding was weaker in the preferred direction compared to the non-preferred direction on delay blocks, and was not significantly different on size blocks for either direction. In the NAc of cocaine-exposed rats we found that reward delivery in the neuron’s preferred direction evoked weaker value encoding. Further, we find that value encoding was overall less directional, in that reward selectivity was similarly encoded in both response directions after cocaine exposure. Lack of directional selectivity in NAc of cocaine-exposed rats is further demonstrated by simply comparing the counts of neurons whose activity—averaged across outcomes on correct trials—was significantly different between directions (Wilcoxon; *p* < 0.05). Rats in the cocaine group had significantly fewer direction-selective neurons during the reward epoch compared to controls (controls: *n* = 53 (27%), cocaine: *n* = 14 (14%) *χ*^2^ = 4.08, *p* < 0.05). Thus, neurons in control rats better differentiated the direction that reward was delivered (i.e., left or right) relative to neurons in rats that had self-administered cocaine.

### **Firing on longer delays was attenuated after cocaine self-administration**

**Fig. 4:**
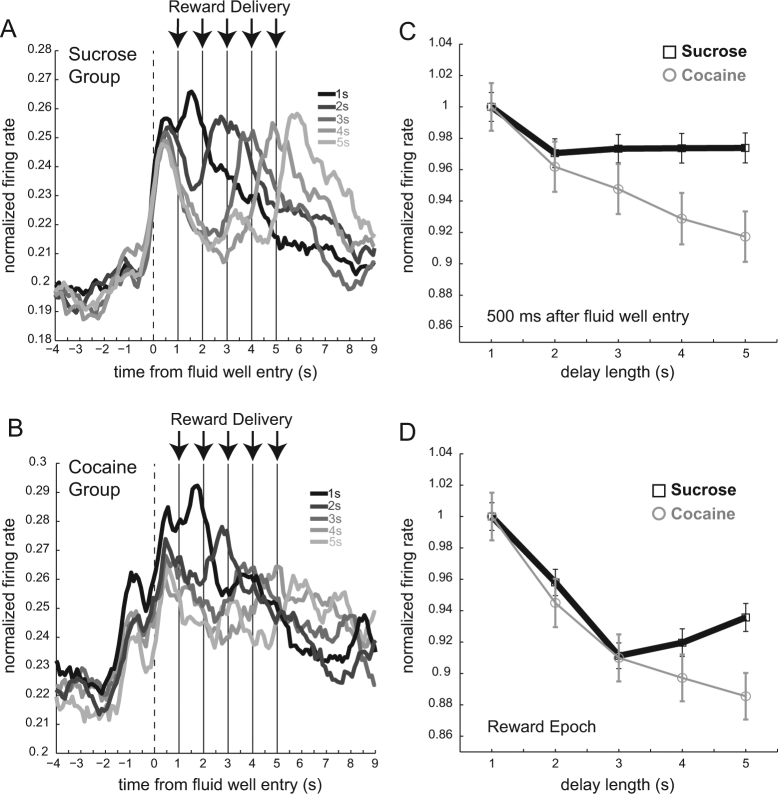
Firing on longer delays was attenuated in cocaine animals. **a** Normalized average firing rates across neurons during reward delivery broken down by delays 1–5 s for control rats (*n* = 195; 6 rats). Activity is aligned to fluid-well entry in order to encompass time immediately upon entry in the fluid well at the start of each delay before reward delivery. Reward delivery is marked by arrows for each delay. Delays 1–5 s are represented by black to lightest gray lines with 1 s delay being black lines and lightest gray lines being 5 s delays. Figure includes both forced and free-choice trials. **b** Normalized average firing rates across neurons during reward delivery broken down by delays 1–5 s for cocaine rats (*n* = 103; 4 rats). Activity is plotted in the same manner as **a**. **c** Normalized average firing rates, divided by maximum firing rate on 1 s delays 500 ms after fluid-well entry. Control rats are plotted in black squares/lines, cocaine rats are plotted in gray circles/lines. Firing rates were normalized to determine how initial firing rates to the shortest long delay declined as they increased in 1 s intervals. **d** Normalized average firing rates, divided by maximum firing rate on 1 s delays during reward delivery epoch (250 ms before reward delivery to 1 s after reward delivery). Control rats are plotted in black squares/lines, cocaine rats are plotted in gray circles/lines

In the sections above, we demonstrate weaker encoding of size and delay at both the population and single neuron level after cocaine exposure. Overall, cocaine rats had fewer neurons that increased firing in anticipation and delivery of reward, and those that were responsive exhibited weaker encoding. Here we ask how firing rates on trials with delayed reward changed as delays got longer. In particular, we were interested in knowing if selectivity in cocaine rats was overly sensitive to longer delays. To address this issue, we plotted firing rates over all neurons that increased firing in both controls and cocaine rats during reward delivery, broken down by delays 1–5 s, aligned to fluid-well entry (6 and 7-s delays were excluded due to insufficient numbers of trials). We examined firing rate at the reward epoch as above, but also analyzed an earlier epoch—500 ms after well entry—that encompassed time immediately upon entry in the fluid well at the start of each delay, without impinging on firing to actual reward delivery (i.e., during the reward epoch). For both epochs, we found that firing declined as delays became longer. This is illustrated in Fig. 4a-b, which represents firing aligned to well entry for delays 1–5 s. Although this decrement was similar for 1–3 s delays in both groups, firing stabilized in control rats during longer delays (Fig. 4a), but continued to decline in rats that had self-administered cocaine (Fig. 4b).

In both groups, firing increased at the time when rats entered the fluid well. Notably this initial response was similar across the 5 delays in controls, but continuously declined in rats that had self-administered cocaine. In controls during this period (500 ms after fluid-well entry), firing rates were not significantly different during delays 2–5 s compared to the 1 s delay (Fig. 4c; Wilcoxons; *p*’s > 0.05). Thus, in controls, after the initial decrease in firing when delays lengthened (i.e., 0.5–1 s), firing at the beginning of the delay, upon well entry, remained stable. This was not true in rats exposed to cocaine in that firing continued to drop as delays became longer; firing was significantly reduced during delays 2–5 s compared to 1 s delay in the cocaine group (Fig. 4c; Wilcoxon; *p* < 0.05).

Consistent with the main analysis described above, both groups showed reduced firing rates for rewards at longer delays; firing to reward during 2–5 s delays was significantly reduced compared to firing during the 1-s delay in both cocaine and control rats (Fig. 4d; Wilcoxon; *p* < 0.05). Also notable is that, during the 5-s delay, firing was significantly higher in controls compared to the cocaine groups (Wilcoxon; *p* < 0.05). Unfortunately, there were not enough trials to adequately determine if this finding persisted through 6 and 7-s delays, thus the significance of this result requires further study.

### Anticipatory firing on errors was not impacted by cocaine

Firing in NAc increases after movements during the anticipation of reward on correct trials. Interestingly, during error trials activity in NAc also increases as if the rat anticipates receiving reward, but then decreases upon the realization that an error has been made and reward would not be delivered [38]. This activity pattern is similar to what occurs on long delay trials described above—there is an increase in firing at the beginning of the trial at the time when reward was expected, but not delivered.

**Fig. 5:**
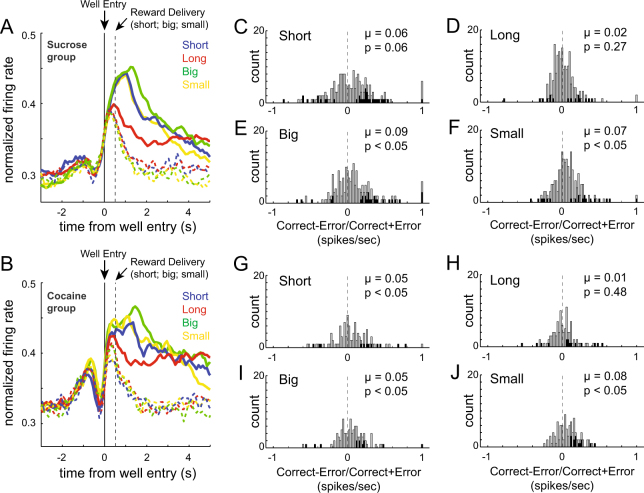
Firing on error trials. **a** Normalized average firing rates across neurons for correct and incorrect trials in the sucrose control group (*n* = 195; 6 rats). Firing is aligned to fluid-well entry in order to encompass activity immediately upon entry in the fluid well at the start of each delay before reward delivery. Reward delivery on correct trials is marked by the gray dashed line for short-delay, large-reward and small-reward trials. Figure includes forced-choice trials only. **b** Normalized average firing rates across neurons for correct and incorrect trials in the cocaine self-administration group (*n* = 103; 4 rats). **c–f** Distribution of error indices for short-delay (**c**), long-delay (**d**), large-reward (**e**), and small-reward (**f**) trials for the sucrose control group. Error index = correct−error/correct + error during the last 250 ms of the 500 ms delay that followed fluid-well entry. Distributions included forced-choice trials only. Black bars represent neurons that showed significant difference between correct and error trials (Wilcoxon, *p* < 0.05). **g–j** Same **c**–**f** except for rats that had self-administered cocaine

In order to determine whether we see similar anticipatory firing when errors were made—and if these signals were altered by prior cocaine self-administration—we generated population histograms for control and cocaine rats on correct and incorrect forced-choice trials. Since expectancy encoding was seen in both directions (Figs. 2 and 3) and errors were less frequent, we averaged across direction to increase the sample size for this analysis. The results are shown in Fig. 5a, b. Note that activity is aligned to well entry (latest possible alignment for error trials because there was no reward) and that on short-delay, small-reward, and large-reward trials, reward was delivered 500 ms after fluid-well entry on correct trials.

As predicted on error trials, firing in NAc increased on all trial types just prior to fluid-well entry as rats moved from the odor port to the fluid-well similar to what can be observed on correct trials (Fig. 5a, b; solid (correct) versus dashed (error)). In our task, houselights were extinguished immediately upon rat entry into an incorrect well. Thus, error commission was cued even prior to the absence of reward. Consistent with this knowledge, anticipatory firing peaked during the 500 ms between well entry and potential reward delivery, and then rapidly declined—resulting in lower firing—prior to the time reward would have been delivered. To quantify this effect, we examined firing during the last 250 ms of the 500 ms period preceding reward delivery and computed an error index (correct−error/correct + error) for the four trial-types (Fig. 5c–j). For both control and cocaine, distributions of error indices were shifted in the positive direction, indicating stronger firing on correct trials prior to reward delivery during performance of short-delay, small-reward and large-reward trial-types (Fig. 5; Cocaine: short-delay, *µ* = 0.05, *p* < 0.05; large-reward, *µ* = 0.05, *p* < 0.05; small-reward, *µ* = 0.08, *p* < 0.05; Control: short-delay, *µ* = 0.04, *p* = 0.06; large-reward, *µ* = 0.09, *p* < 0.05; small-reward, *µ* = 0.07, *p* < 0.05). When directly comparing the error index distributions between control and cocaine rats, we found that there were no significant differences between groups (Wilcoxon rank sum, *p*’s > 0.05).

Unlike the other trial types, for long-delay trials, the distributions for cocaine and control rats were not significantly shifted from zero (Wilcoxon: Fig. 5h, Cocaine, *µ* = 0.01, *p* = 048; Fig. 5d, Control, *µ* = 0.02, *p* = 0.27). Cocaine and control error distributions did not significantly differ from each other (Wilcoxon; *p* = 0.97). The lack of difference in firing between correct and error trials early during long delays is not surprising considering that the anticipated reward was not delivered 500 ms after reward delivery in either situation. That is—during an error, reward was omitted due to an incorrect response and during correct long-delay trials, rewards were not delivered after 500 ms but delayed by several seconds. Overall, these results demonstrate that neurons in NAc fire in anticipation of reward on error trials and that this signal is unaffected by prior cocaine exposure.

### Outcome selectivity during the odor epoch was not altered by cocaine

Above we showed that neural encoding during the reward epoch was affected by cocaine self-administration. Here, we perform the same analysis but during the period between odor onset and well entry (i.e., odor epoch = 100 ms after odor onset to fluid-well entry). In control and cocaine-exposed rats, 175 (27%) and 99 (20%) neurons increased firing during the odor cue period compared to baseline (1 s before odor onset; Wilcoxon, *p* < 0.05), respectively. The frequency of responsive neurons was significantly different between groups, with more neurons in NAc increasing firing during the odor epoch in controls compared to cocaine rats (controls *n* = 175, cocaine *n* = 99, *χ*^2^ = 3.92, *p* < 0.05). As above, we determined how many of these neurons were directionally selective by comparing firing on left versus right trials averaged across different outcomes (Wilcoxon; *p* < 0.05). The counts of neurons that fired significantly differently between left and right directions did not significantly differ between control and cocaine groups (Wilcoxon; *p* < 0.05; controls *n* = 18, cocaine *n* = 20, *χ*^2^ = 3.18, *p* = 0.07). Finally, there were no differences in counts of neurons that encoded predicted outcomes (short, long, big, and small) in the preferred direction (Wilcoxon; *p* < 0.05) during delay blocks (controls: short *n* = 8, long *n* = 8; cocaine: short *n* = 13, long *n* = 3; *χ*^2^ = 2.22, *p* = 0.13) or size blocks (controls: big *n* = 20, small *n* = 10; cocaine: big *n* = 11, small *n* = 5; *χ*^2^ = 0.03, *p* = 0.85). Thus, overall, we conclude that although there were significantly fewer counts of neurons responsive prior to well entry during odor presentation and the decision to move, there were no significant differences between controls and cocaine rats in what was encoded during this period.

## Discussion

The NAc is thought to be one of the first brain regions to be affected by drug abuse [39–48]. Here, we examined how previous cocaine self-administration impacts neural correlates involved in reward expectancy and valuation. We achieved this by analyzing neural activity in the NAc while rats performed a reward-guided decision-making task in which the value of reward was altered through independent manipulations of the delay to and size of reward. We show that cocaine exposure results in response bias toward shorter delays to reward, faster overall responding, and a reduction in value and direction encoding in NAc at the single neuron and population level.

### Cocaine increases impulsive choice during delay discounting

As described in several previous studies, rats previously exposed to cocaine more strongly biased behavior to rewards delivered after a shorter delay [30–35]. Although we replicated the result that cocaine made rats more impulsive on delay trials, unlike our previous publications, we did not observe significant changes in behavior during manipulation of reward size [35, 49]. It is unclear why these differences have emerged, but in part they may be attributable to variations in self-administration paradigms. In the original 2007 paper, cocaine was administered by the experimenter and neural recordings were not performed. In our most recent publication, rats self-administered cocaine but not all of the controls self-administrated sucrose. Here, rats self-administered cocaine and all control rats self-administered sucrose pellets. Future work is necessary to determine if route of sucrose and cocaine administration impacts behavioral effects and why we see neural alterations in size processing in NAc that do not translate behaviorally. Nevertheless, we clearly replicate the result that cocaine exposure increases sensitivity to delay manipulations across all three studies. Notably, the majority of work outside our lab that has examined the impact of drugs of abuse on delay discounting has shown effects on delay but not size, suggesting that behavioral selection related to delay processing is more susceptible to cocaine exposure [31, 34].

### **Impact of cocaine on NAc firing and downstream areas**

Consistent with cocaine’s impact on behavior, we saw several alterations in NAc that might contribute to abnormal task performance. First, we observed an overall reduction in the number of cells responsive to both odor cues and reward delivery. Second, in those cells that remained reward responsive, we observed a reduction in direction and value encoding. Third, neural firing to delayed rewards was weaker in rats that had self-administered cocaine.

Although altered and lost directional and value encoding in the NAc could affect several different downstream systems, we speculate that the behavioral and neurophysiological results described here are tightly linked to changes in dopamine (DA) signaling. It has recently been shown that reward prediction errors signaled by dopaminergic neurons in the VTA depend upon accurate signaling from the NAc, specifically in relation to the timing of rewarded outcomes [29] and that this is essential for developing neural selectivity to cues in DA neurons and neural signaling in the NAc [27, 50]. We suspect that diminished expectancy and reward encoding in NAc results in reduced cue-evoked DA release and enhanced prediction error signals, which likely lead an increased propensity to seek more immediate reward in our task.

Several studies have shown changes in DA signaling in NAc after cocaine exposure [51–53]. Specifically, cocaine exposure has been shown to diminish both neural activity in the NAc and DA release in the NAc to cues predicting rewards and punishments during reversal and Pavlovian discrimination tasks [54–56]. Taken together, these findings fit with our overall result of fewer task responsive neurons in the NAc after cocaine exposure, which ultimately could impact the processing of cues, rewards, and associated actions in downstream areas.

Consistent with this idea, we have shown that lesions to NAc and exposure to cocaine impacts encoding in DLS [28, 56], albeit in different ways. After NAc lesions, neural correlates related to stimulus and response encoding in DLS were enhanced. After cocaine self-administration, stimulus–response correlates were unaltered, whereas correlates related to response-outcome encoding were enhanced and divorced from actions [35]. That is, after cocaine exposure, correlates in DLS better reflected the contingencies available during decision making as opposed to a representation of what would ultimately be selected. Notably, these correlates were amplified after chronic cocaine self-administration at the expense of correlates that signal the outcome predicted by what the actual decision will be. This lack of processing of direction and value in DLS might have resulted from the impaired encoding in NAc described here. Specifically, we think that since NAc is no longer accurately signaling the direction of the reward obtained and its expected and actual value, this information is no longer being transmitted to the DLS [64–68]. Without this information, DLS signals are swayed by action-outcome information regarding the response direction associated with different rewards, not the reward that would actually be delivered based on the response that was selected, possibility leading to failed modification of online reward predictions.

## Conclusion

We conclude that previous cocaine self-administration has a long-term impact on decision making and signaling in the NAc during performance of a complex behavioral decision-making task that independently varies the size of and delay to reward. We suspect that other signals related to cost, risk, and effort discounting in NAc are likely to be impacted by cocaine self-administration, but this has yet to be determined [57–63]. Overall, cocaine exposure resulted in a behavioral bias toward shorter delays and a reduction in the amount of NAc cells that responded to odor cues and expected rewards. In addition, the reward-responsive cells that remained after cocaine exposure showed diminished value and directional selectivity. Reduced encoding of value and direction suggests that prior cocaine self-administration would impair the ability of NAc to guide behavior via model-based mechanisms that are reliant on this information to normally guide decision making.
